# Draft Genome Sequence of the Symbiotic *Frankia* sp. strain B2 isolated from root nodules of *Casuarina cunninghamiana* found in Algeria

**DOI:** 10.7150/jgen.38461

**Published:** 2020-01-19

**Authors:** Kathia Belaid, Erik Swanson, Alyssa Carré-Mlouka, Valérie Hocher, Sergio Svistoonoff, Djamel Gully, Stephen Simpson, Krystalynne Morris, W. Kelley Thomas, Said Amrani, Louis S. Tisa, Hassen Gherbi

**Affiliations:** 1 Laboratoire de Biologie du Sol, Faculté des Sciences Biologiques, Université des Sciences et de la Technologies Houari Boumediene (USTHB), BP32 El Alia - Bab Ezzouar Algiers, Algeria.; 2 University of New Hampshire, 46 College Rd., Durham, New Hampshire, USA, 03824-2617; 3 Laboratoire des Symbioses Tropicales et Méditerranéennes (IRD/INRA/CIRAD/Université de Montpellier/Supagro), 34398 Montpellier Cedex 5, France.; 4 Laboratoire Molécules de Communication et Adaptation des Microorganismes (MCAM) UMR 7245 CNRS-MNHN), Museum national d'Histoire naturelle, Centre National de la Recherche Scientifique (CNRS), CP 54, 57 rue Cuvier, 75005 Paris, France

**Keywords:** actinorhizal symbiosis, host-microbe interactions, nitrogen fixation, *Casuarinaceae*, * Frankia*, land reclamation, genomes

## Abstract

*Frankia* sp. strain B2 was isolated from *Casuarina cunninghamiana* nodules*.* Here, we report the 5.3-Mbp draft genome sequence of *Frankia* sp. strain B2 with a G+C content of 70.1 % and 4,663 candidate protein-encoding genes. Analysis of the genome revealed the presence of high numbers of secondary metabolic biosynthetic gene clusters.

## Introduction

Actinobacteria of the genus *Frankia* are Gram positive filamentous bacteria that are able to fix molecular nitrogen in free living state or in symbiosis with their host plant 1, 2. These bacteria establish a nitrogen-fixing symbiosis with a diverse variety of plant species, collectively named actinorhizal plants, which include 8 dicotyledonous plant families, 24 genera and over 220 species. The mutualistic association is referred to as the actinorhizal symbiosis and results in the formation of a root nodule structure. The bacteria are housed within plant cells in the nodule which allows for the trophic exchange between the two partners. The bacteria reduce atmospheric nitrogen to ammonia that is supplied to the host plant, which in return provides carbon compounds from photosynthesis to the bacteria. Because of the symbiosis, actinorhizal plants can colonize poor and degraded soils and thrive in inhospitable and harsh habitats 2. Actinorhizal plants are pioneer species that allow the succession of other plant communities by providing organic matter, a fundamental matrix for the dynamics and biodiversity of terrestrial ecosystems. There is currently a renewing interest for actinorhizal symbiosis due to its significant contribution to global soil amendment in combined nitrogen (more than 15%) 3.

Based on the recent molecular phylogenetic studies, *Frankia* strains are classified into four major clusters 4-6 that reflect host plant range. Cluster 1 consists of *Frankia* strains that associate with host plants in the Casuarinaceae, Betulaceae and Myricaceae families, while members of cluster 2 are infective on Rosaceae, Coriariaceae, Datiscaceae, and the genus *Ceanothus* (Rhamnaceae). Cluster 3 are the most promiscuous and are infective on Elaeagnaceae, Rhamnaceae, Myricaceae, *Gynmnostoma*, and occasionally the genus *Alnus.* Cluster 4 consists of “atypical” *Frankia* strains that are unable to re-infect actinorhizal host plants or form ineffective nonnitrogen-fixing root nodule structures. Cluster 1 is further divided into subclades. Subclade Ic includes strains limited to *Casuarina* and *Allocasuarina* and Myricaceae host plants.

Actinorhizal species include *Casuarina* spp., tropical trees native in Australia, Southeast Asia and Oceania 7. These woody plants are well adapted to drought, heat, salinity, polluted soils and can withstand multiple varieties of environments 2. This property is one reason why they have been massively planted in several regions of the globe for land reclamation, prevention of erosion, crop protection and fighting against desertification, tsunamis and typhoons 7. In Algeria, like in all the Maghreb, *Casuarina* trees were introduced in the 19^th^ century and are currently found widespread in all bioclimatic zones of the country ranging from the coastal zone to the Saharan areas. Today, the propagation of *Casuarina* trees occurs mostly from plantlets produced in nurseries *via* seeds or by cutting. As a part of a project that aims to reassess the identity, the distribution and the relative abundance of *Casuarina* trees in Algeria, we were interested in investigating the prevalence of actinorhizal symbiosis in nurseries from different regions of the country, and to examine whether the symbiotic status can help the installation of the plantlets in natural environments. For this purpose, we have collected nodules samples from young *Casuarina* trees from Algerian nurseries and the symbiotic *Frankia* strain was isolated.

## Isolation of *Frankia* strain B2

**Figure 1:**
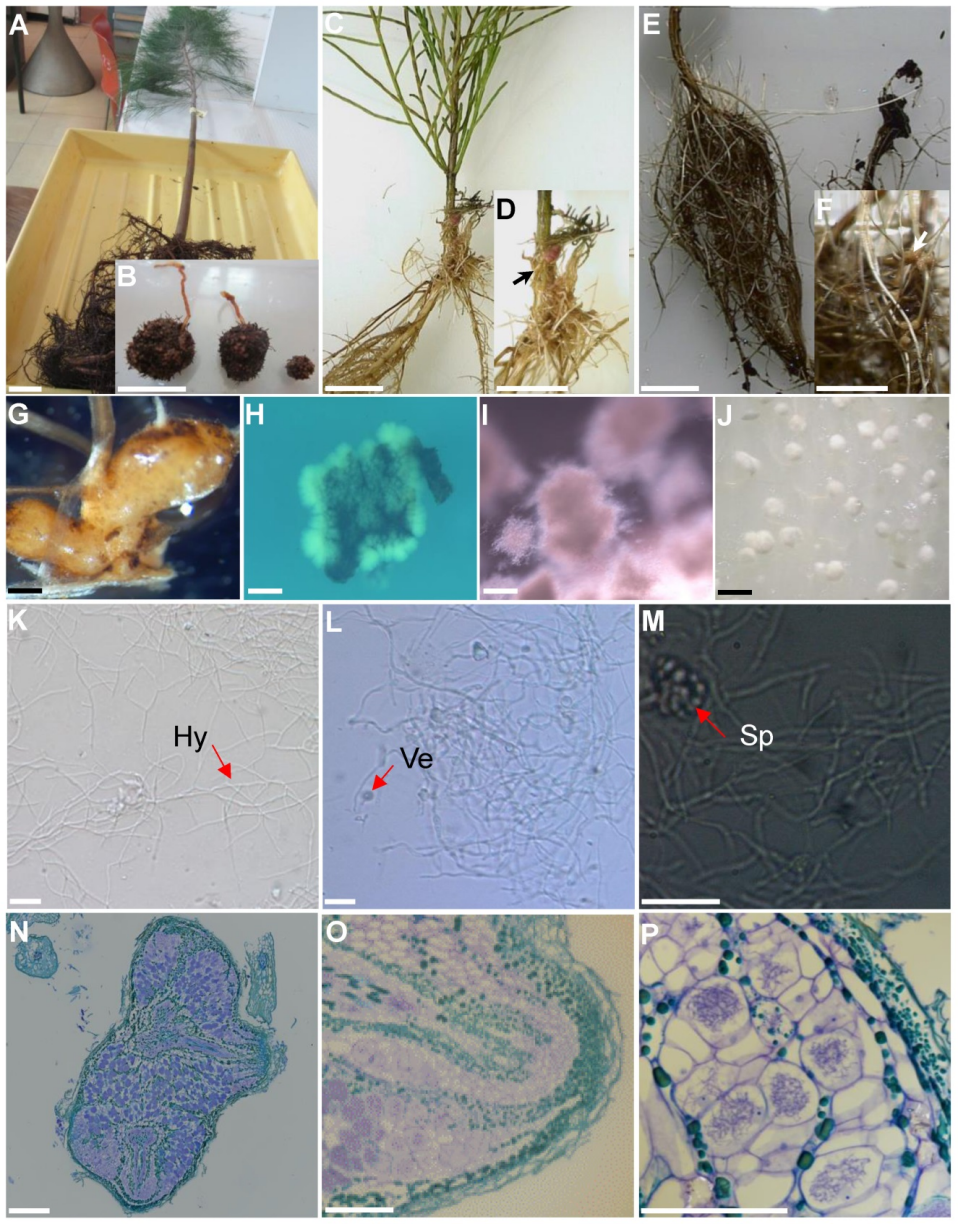
** Isolation of *Frankia* strain B2. (A)** A nodulated young *Casuarina cunninghamiana* grown in a nursery of Souk El Tenine (District of Bejaia, Algeria). **(B)** *C. cunninghamiana* root nodules. **(C)** Nodulation of *C. cunninghamiana* after inoculation with crushed nodules previously harvested from *C. glauca* growing hydroponically in N-free BD medium (8) for 8 weeks in growth chamber at 25° C with 75% of relative air humidity and 16 h of photoperiod. **(D)** Close up of C, showing nodule (arrow). **(E)** *C. glauca* nodulation after inoculation with crushed nodules harvested from *C. cunninghamiana* grown in the nursery. **(F)** Close up of panel E, showing nodule (arrow). **(G)** *Casuarina glauca* young nodule used for *Frankia* B2 isolation. **(H)** Development of *Frankia* B2 from a surface-sterilized *C. glauca* nodule fragment cultivated on BAP solid medium. **(I)** Cultures of *Frankia* B2 grown in BAP liquid medium. **(J)** *Frankia* B2 colonies cultivated on BAP solid medium. **(K)** *Frankia* B2 isolated from colonies (see panel J) and cultivated in BAP liquid medium. Hyphae are observed (Hy). **(L, M)** *Frankia* B2 liquid culture showing vesicles (Ve) and sporangia (Sp). **(N)** Longitudinal section of a *C. cunninghamiana* nodule stained with toluidine blue. **(O)** Close up of panel N showing a nodule lobe. **(P)** Close up of panel O showing cortical infected cells. Scale bars: A-F = 20 mm; G-J = 5 mm; K-M = 100 µm; N-O = 100 µm.

**Figure 2:**
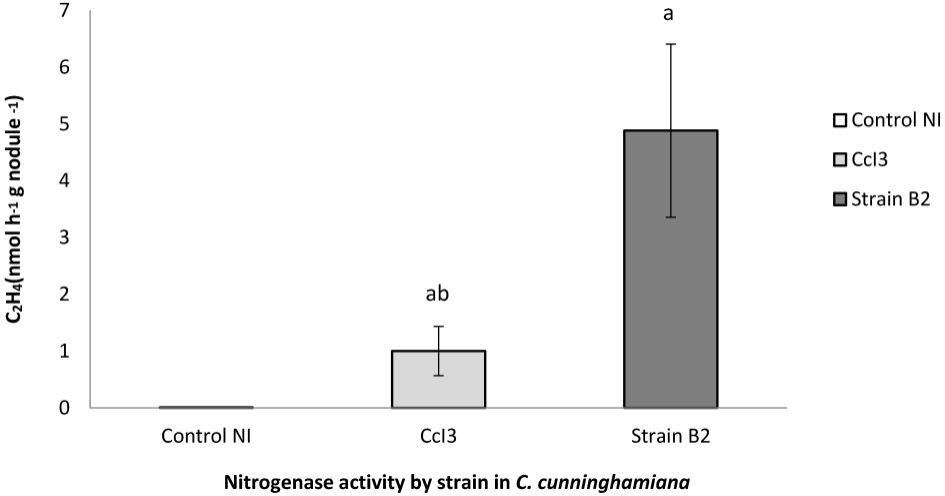
** Nitrogenase activity of nodules induced by *Frankia* strain B2.** The acetylene reduction activity (ARA) was used to determine nitrogenase activity of *C. cunninghamiana* 14*.* Nodules of *C. cunninghamiana* were induced with *Frankia* strain B2 or* F. casuarinae* strain CcI3. The uninoculated *C. cunninghamiana* plants (NI) were included as a control. Values represent the mean of several measurements (Control NI, N = 4; *F. casuarinae* strain CcI3, N = 4; and *Frankia* strain B2, N = 23), Error bars indicate standard error of the mean ANOVA-LSD analysis shows that a significant difference among the samples.

*Frankia* strain B2 was isolated in two-step process from nodules collected from *Casuarina cunninghamiana* seedlings growing in a nursery located at Souk El Tenine (District of Bejaia, Algeria). For the first step, the collected nodules were crushed and used as an inoculum on *Casuarina glauca* plants growing hydroponically in N-free BD medium 8 in a culture chamber under controlled conditions (25° C, 75% of relative air humidity and 16 h of photoperiod. After 8 weeks, root nodules were observed and harvested. For the second step, harvested nodules were washed, fragmented and surface-sterilized by immersion in a 30% H_2_0_2_ solution for 30 min based on protocol described previously 9. Sterilized nodule fragments were inoculated onto the surface of different solid growth media including BAP 10, DPM (Defined Propionate Minimal Medium) 11 or modified QMOD 12 under nitrogen-free conditions (without yeast extract and peptone for QMOD). Plates were incubated in dark at 28°C. After 4-6 weeks, *Frankia* hyphae developed around the nodule fragments inoculated on BAP medium and these colonies were transferred into liquid BAP growth medium. Figure 1 shows the different stages of the isolation process and photomicrographs show typical *Frankia* features. *Frankia* has three different morphogenetic forms; vegetative hyphae (Hy), vesicles (Ve), the site of nitrogen fixation and sporangia containing spores (Sp). All three types of cell structures were produced by *Frankia* strain B2 (Fig. 1K-M). *Frankia* strain B2 was able to re-infect *C. cunninghamiana* and the nodules produced (Fig. 1N-O) showed a higher level of nitrogenase activity compared to *C. cunninghamiana* nodules with *Frankia casuarinae* strain CcI3, the type strain 13 (Fig. 2). The acetylene reduction activity (ARA) was used to determine nitrogenase activity of *C. cunninghamiana* 14. Because *Frankia* strain B2 had these traits and it represented an Algerian isolate, we chose to sequence its genome.

## Sequencing of *Frankia* strain B2

Sequencing of the draft genome of *Frankia* sp. strain B2 was performed at the Hubbard Center for Genome Studies (University of New Hampshire, Durham, NH) using Illumina technology techniques 15. High quality gDNA of *Frankia* sp. strain B2 was extracted using CTAB method 16. A standard Illumina shotgun library was constructed and sequenced using the Illumina HiSeq2500 platform, which generated 4,247,110 reads (260-bp insert size) totaling 965 Mbp. The Illumina sequence data were trimmed by Trimmomatic version 0.36 17, and assembled using Spades version 3.10 18. The final draft assembly for *Frankia* sp. strain B2 consisted of 145 contigs with an N_50_ contig size of 103.6 kb and 176X coverage of the genome. The final assembled genome contained a total sequence length of 5,331,433 bp with a G+C content of 70.12%.

The assembled *Frankia* sp. strain B2 genome was annotated *via* the NCBI Prokaryotic Genome Annotation Pipeline (PGAP), and resulted in 4,663 candidate protein-encoding genes, 41 tRNA and 5 rRNA. The genome features of *Frankia* sp. strain B2 are similar to other cluster 1c genomes (Table 1) including *F. casuarinae* strain CcI3^T^ 13. Phylogenetic analysis of the 16S rDNA shows that *Frankia* sp. strain B2 groups with the cluster 1c strains (Figure S1) and further confirmed by dendrogram of the entire genomes (Figure S2). The genome also contained a *nif,* 2 *hup,* and 1 *shc* operons encoding the nitrogenase, hydrogenase uptake enzymes, and the hopanoid biosynthetic pathway, respectively. The operons were organized similar to those reported for *Frankia* cluster 1c genomes 19. The pan-genome of *Frankia* cluster 1c consisted of 4,736 genes including a core genome of 3,107 genes. Figure S3 shows a Venn diagram of the orthologs shared among six *Frankia* cluster 1c strains.

Bioinformatic analysis of this genome by the use of the AntiSMASH program 20 revealed the presence of high numbers of secondary metabolic biosynthetic gene clusters, which is consistent with previous results for other *Frankia* genomes including subcluster Ic 19, 21. Table 2 shows a comparison of the various profiles of different *Casuarina* isolates for these secondary metabolic biosynthetic gene clusters. Although the majority of these secondary metabolic biosynthetic gene clusters were shared among the *F. casuarinae* genomes, the *Frankia* sp. strain B2 genome contained five unique nonribosomal peptide synthase (NRPS) clusters that were completely novel without homologues to other microbes but had minimal information on the chemical structures of the natural products. Predicted monomers for some of these unique NRPS clusters were identified, but no structure could be predicted from this algorithm.

In summary, the *Frankia* sp. strain B2 genome has revealed an interesting potential for secondary metabolites pathways and natural product profile and serves as another representative of *Frankia* cluster 1c.

### Nucleotide sequence accession numbers

This whole-genome shotgun sequence has been deposited at DDBJ/EMBL/GenBank under the accession number SOPN00000000.1. The version described in this paper is the first version, SOPN01000000.

## Supplementary Material

Supplementary figures.

## Figures and Tables

**Table 1:** Genome features of *Frankia* sp. strain B2 and other *Frankia* strains isolated from *Casuarina* root nodules.

Strain	Source	Location^1^	Size (Mb)	No. of Contig(s)	G+C (%)	No. of CDS	No. of rRNA	No. of tRNA
B2	This study	Algeria	5.33	145	70.1	4,663	5	45
KB5	22	Australia	5.46	420	70.0	4,958	6	45
CcI3	23	USA	5.43	1	70.1	4,598	6	46
CeD	24	Senegal	5.00	120	70.1	4,403	7	45
Allo2	25	Uruguay	5.33	110	69.8	4,838	7	46
Thr	26	Egypt	5.31	171	70.0	4,805	5	46
BMG5.23	27	Tunisia	5.27	167	70.0	4,747	9	47
CcI6	28	Egypt	5.39	138	67.6	4,902	9	46
BR	29	Brazil	5.23	180	70.0	4,777	5	46

^1^ The source of the isolate.

**Table 2:** Biosynthetic gene clusters for natural products found in the genomes from *Casuarina Frankia* strains.

Strain	No. of Biosynthetic gene clusters ^1^	NRPS ^2^	PKS ^3^	Terpene	Siderophore	Bacteriocin	Lantipeptide
B2	31	6	9	4	1	0	6
KB5	34	4	9	6	1	1	4
CcI3	29	3	5	4	1	3	6
CeD	30	7	7	4	1	1	4
Allo2	32	7	9	4	1	3	5
Thr	33	6	7	4	1	1	6
BMG5.23	31	8	6	4	1	2	4
CcI6	33	8	8	4	1	3	5
BR	29	5	5	4	1	2	5

^1^Biosynthetic gene clusters were identified by the use of the AntiSMASH software^30^, ^31^. ^2^NRPS: Nonribosomal peptide synthase. ^3^PKS: polyketide synthase including Type I, II, III, Trans-AT, and other types
