# Supplementary material for: Draft Genome Sequence of the Symbiotic Frankia sp. strain B2 isolated from root nodules of Casuarina cunninghamiana found in Algeria
Source: J Genomics. 2020 Jan 19;8:11–5. doi: 10.7150/jgen.38461 (PMC7019079; doi:10.7150/jgen.38461)
Supplement: Supplementary file 1 — Supplementary figures. [file jgenv08p0011s1.pdf]

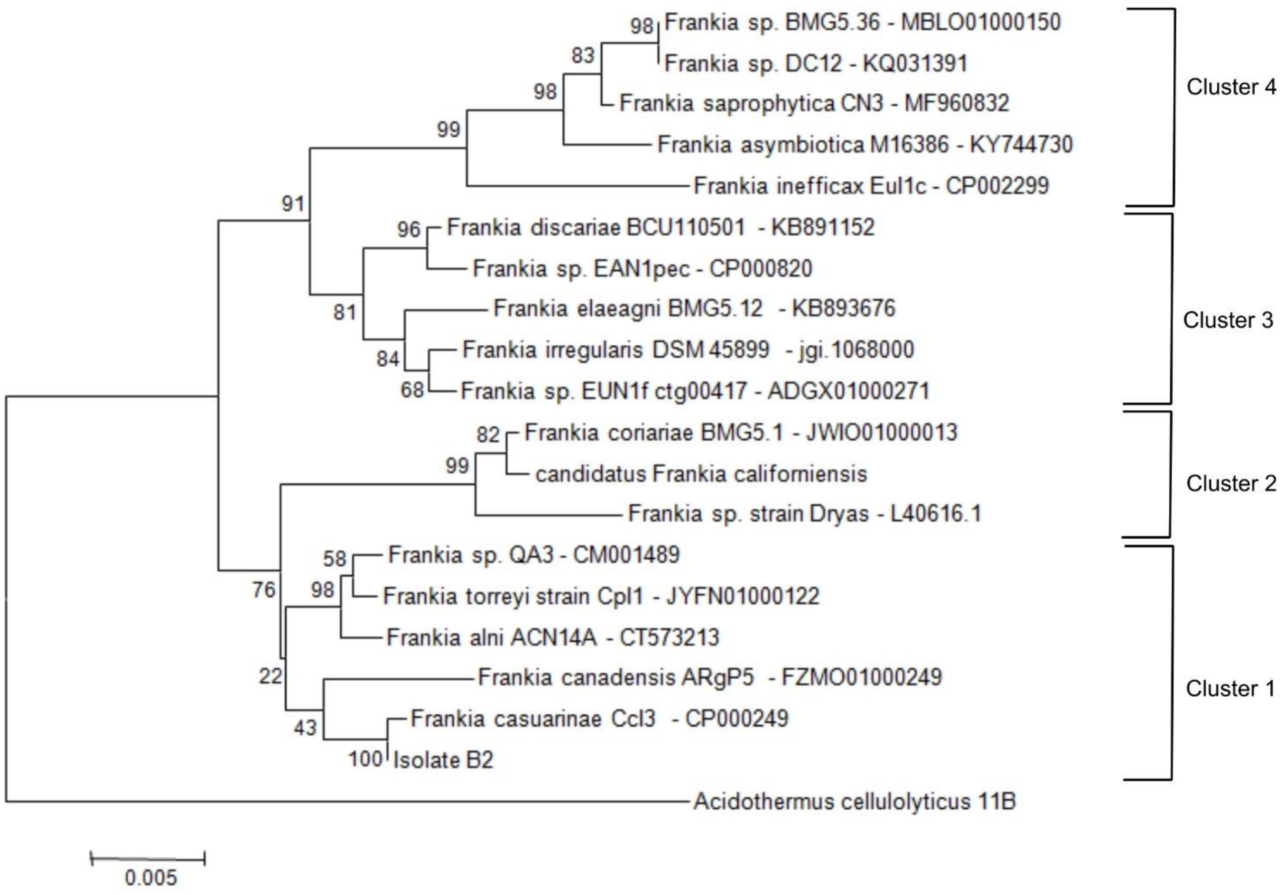

Figure S1. Evolutionary relationships of taxa. The evolutionary history was inferred using the Neighbor-Joining method [1]. The optimal tree with the sum of branch length = 0,13081716 is shown. The percentage of replicate trees in which the associated taxa clustered together in the bootstrap test (1000 replicates) are shown next to the branches [2]. The tree is drawn to scale, with branch lengths in the same units as those of the evolutionary distances used to infer the phylogenetic tree. The evolutionary distances were computed using the Maximum Composite Likelihood method [3] and are in the units of the number of base substitutions per site. The analysis involved 20 nucleotide sequences. All positions containing gaps and missing data were eliminated. There were a total of 1268 positions in the final dataset. Evolutionary analyses were conducted in MEGA6 [4] by using *Acidothermus cellulolyticus* strain 11B (= ATCC 43068) as an outgroup.

References

1. Saitou N, Nei M. The Neighbor-Joining Method - a New Method for Reconstructing Phylogenetic Trees. Mol Biol Evol. 1987; 4: 406-25.
2. Felsenstein J. Evolutionary Trees from Gene-Frequencies and Quantitative Characters - Finding Maximum-Likelihood Estimates. Evolution. 1981; 35: 1229-42.
3. Tamura K, Nei M, Kumar S. Prospects for inferring very large phylogenies by using the neighbor-joining method. P Natl Acad Sci USA. 2004; 101: 11030-5.
4. Tamura K, Stecher G, Peterson D, Filipski A, Kumar S. MEGA6: Molecular Evolutionary Genetics Analysis Version 6.0. Mol Biol Evol. 2013; 30: 2725-9.

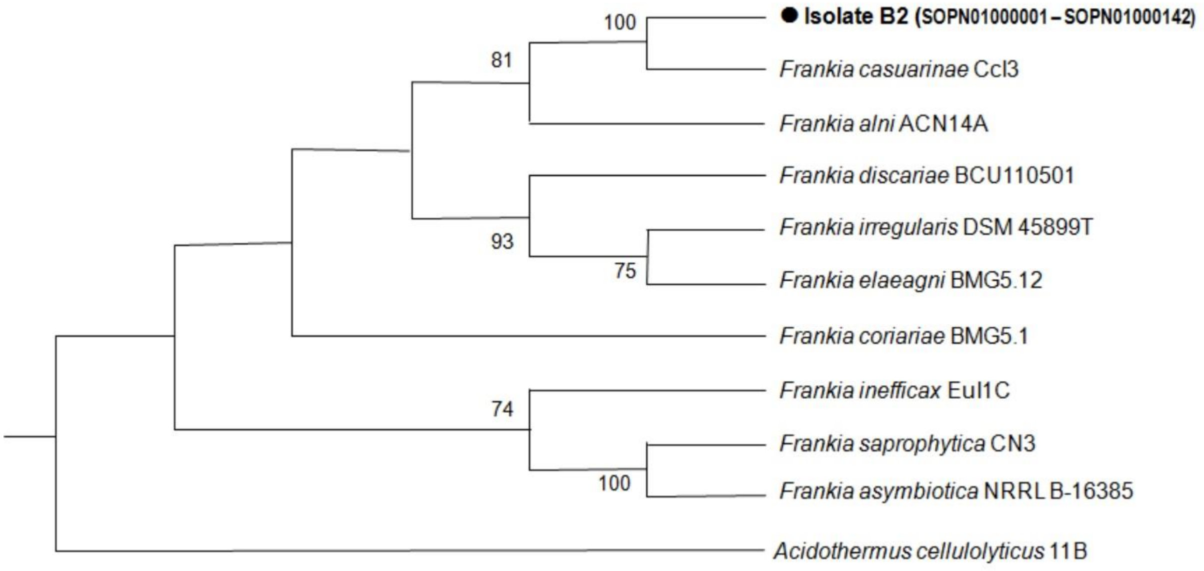

Figure S2. Tree inferred with FastME 2.1.6.1 [1] from GBDP distances calculated from genome sequences. The branch lengths are scaled in terms of GBDP distance formula d_5. The numbers above branches are GBDP pseudo-bootstrap support values from 100 replications, with an average branch support of 74.5 %. The tree was rooted at the midpoint [2].

1. Lefort V, Desper R, Gascuel O. FastME 2.0: A Comprehensive, Accurate, and Fast Distance-Based Phylogeny Inference Program. Mol Biol Evol. 2015; 32: 2798-800.
2. Farris JS. Estimating Phylogenetic Trees from Distance Matrices. Am Nat. 1972; 106: 645-&.

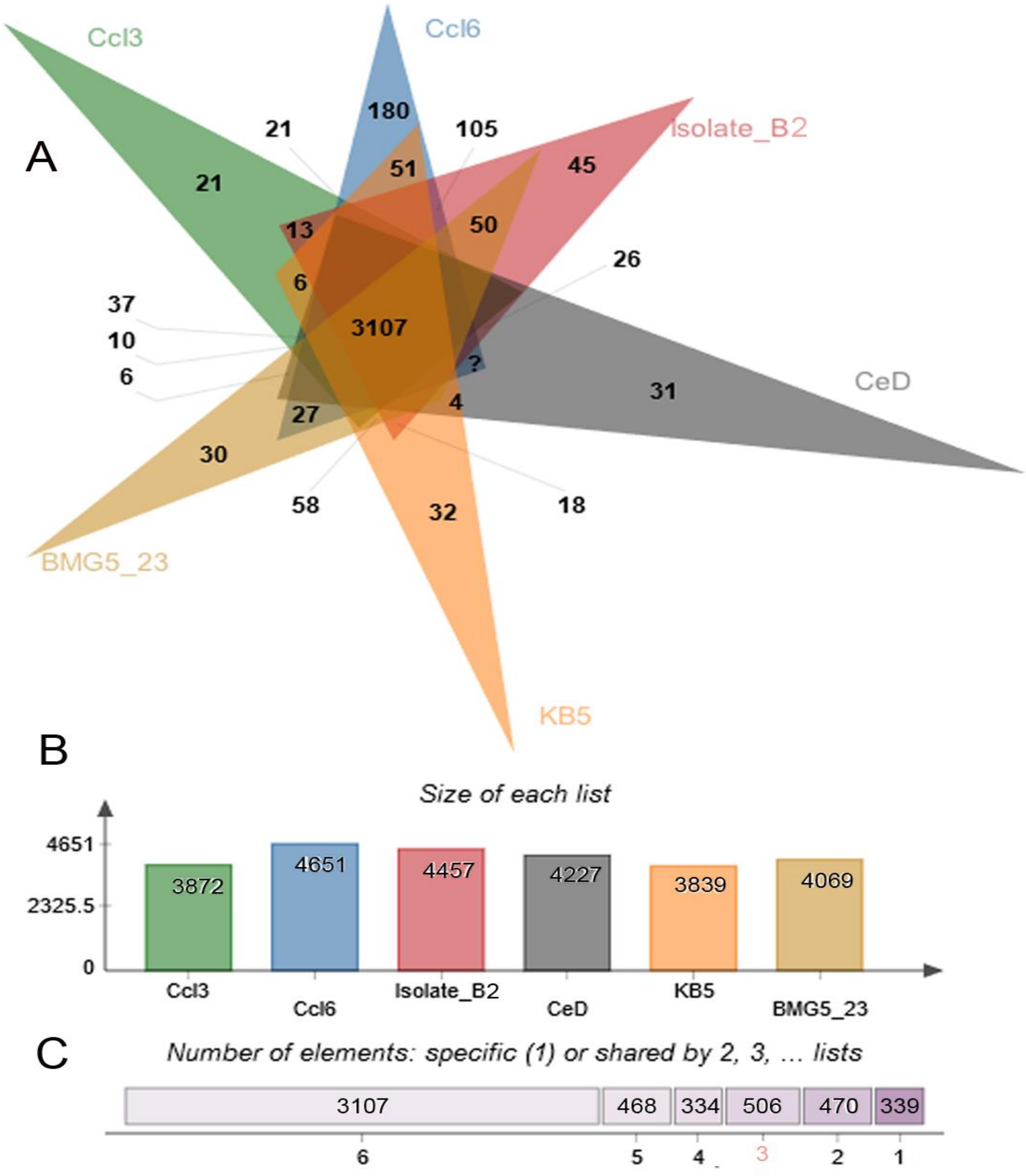

Figure S3 Comparative genomics among the six Cluster Ic genomes. **a.** Venn diagram of the shared and unique orthologs among the six Cluster Ic genomes. **b.** Totals of orthologs in each genome that were used to generate the Venn diagram. **c.** Number of shared orthologs among 6, 5, 4, 3, 2 or 1 genome.
